# Evaluation of the self-reported questionnaires used to assess mental health after the January 2015 terrorist attacks in the Paris Region: IMPACTS survey

**DOI:** 10.1192/j.eurpsy.2022.673

**Published:** 2022-09-01

**Authors:** L. Bertuzzi, C. Vuillermoz, T. El Aarbaoui, M. Héron, L. Aubert, P. Pirard, S. Vandentorren, Y. Motreff

**Affiliations:** 1 Sorbonne Université, Inserm, Institut Pierre Louis D’épidémiologie Et De Santé Publique, Iplesp, Social Epidemiology Research Team, Paris, France; 2 Santé publique France, Cire Antilles, Pointe-à-pître, France; 3 Santé publique France, Direction Des Maladies Non Transmissibles Et Traumatismes, Saint Maurice, France; 4 Santé publique France, Direction Scientifique Et Internationale, Saint Maurice, France

**Keywords:** Mental health disorders, self-report questionnaires, Terrorist attack

## Abstract

**Introduction:**

Structured clinical interviews are the gold standard for assessing mental health. However limited resources may allow the use of only self-report questionnaires. In the context of emergency, such as terrorist attacks, the performance and thresholds of such tools still unclear.

**Objectives:**

We investigated the performance of the Posttraumatic stress disorder CheckList Scale (PCL-S) and of the Hospital Anxiety and Depression scale (HADS), both compared to the MINI Interview, among civilians and first responders involved in terrorist attacks.

**Methods:**

The data came from the IMPACTS survey which was conducted from 6-10 months among civilians (N=190) and first responders (N=232) after the January 2015 terrorist attacks in the Paris Region, France. Sensitivity and specificity of the PCL-S and HADS were estimated by the ROC curve, and the optimal threshold was defined using the Youden index.

**Results:**

Regarding the PCL-S: for civilians and first responders respectively, the overall AUC was 0.947 and 0.899, and the optimal threshold were 38.5 and 39.5. Regarding the HADS-D: for civilians and first responders respectively, the overall AUC was 0.908 and 0.617 and the optimal thresholds were 7.5 and 1.5. For the HADS-A for civilians and first responders respectively, the overall AUC was 0.823 and 0.717, the optimal threshold were 9.5 and 6.5.

**Conclusions:**

In the context of a terrorist attack, compared to the MINI, our study underlined satisfactory performance of the PCL-S and the HADS-D in screening for PTSD and depression respectively, while the screening of anxiety using the HADS-A was unsatisfactory.

**Disclosure:**

No significant relationships.

